# Morphological and Molecular Evidence Revealed New Species in *Mactra antiquata* Sensu Lato

**DOI:** 10.3390/biology15020178

**Published:** 2026-01-18

**Authors:** Fei Teng, Peizhen Ma, Yang Zhang, Jiazhen Zhang, Yuan Zhang, Jie Liu, Haiyan Wang

**Affiliations:** 1 National Maritime Museum of China, Tianjin 300450, China; ghb_keyan@163.com (F.T.); chief00222@163.com (Y.Z.); zhangjiazhenfio@163.com (J.Z.); julieleta@outlook.com (Y.Z.); 2 State Key Laboratory of Mariculture Biobreeding and Sustainable Goods, Yellow Sea Fisheries Research Institute, Chinese Academy of Fishery Sciences, Qingdao 266071, China; mapz@ysfri.ac.cn; 3 Laboratory for Marine Fisheries Science and Food Production Processes, Qingdao Marine Science and Technology Center, Qingdao 266237, China; 4 Laboratory of Marine Organism Taxonomy & Phylogeny, Shandong Province Key Laboratory of Marine Biodiversity and Bio-Resource Sustainable Utilization, Institute of Oceanology, Chinese Academy of Sciences, Qingdao 266071, China

**Keywords:** cryptic species, mitochondrion, morphological characteristic, identification

## Abstract

*Mactra antiquata* sensu lato, a commercially important clam species in China, exhibits remarkable morphological and molecular diversity, which has led to the proposal of cryptic species within this complex. In the present study, specimens of *M. antiquata* sensu lato were collected from four coastal provinces (Shandong, Guangdong, Guangxi, and Hainan) of China, and an integrated comparative analysis was performed based on morphological traits and partial sequences of two mitochondrial genes (cytochrome c oxidase subunit I, *COI*; and *16S* rRNA). Our results revealed that *M. antiquata* sensu lato could be clearly delineated into two distinct clades: the N-group (comprising specimens collected from Shandong in this study) and the S-group (including specimens collected from Guangdong, Guangxi, and Hainan), with significant intergroup differences. Morphologically, S-group individuals possessed relatively narrower shells (mean shell width-to-length ratio = 0.465) and shorter shells (mean shell height-to-length ratio = 0.781) compared to N-group conspecifics. Additionally, the pallial sinus of S-group clams extended directly toward the anterior adductor muscle, whereas that of N-group clams pointed to the region below the anterior adductor muscle. Furthermore, the escutcheon of N-group individuals was considerably more slender than that of the S-group. Phylogenetic trees and haplotype networks constructed based on both partial *COI* and *16S* rRNA sequences further confirmed a deep genetic divergence between the two groups, with Kimura 2-parameter distances of 0.158 for *COI* and 0.084 for *16S* rRNA. Collectively, these morphological and molecular lines of evidence strongly support the existence of cryptic species within *M. antiquata* sensu lato. By comparing the morphological characteristics of specimens in this study with the original description of *M. antiquata*, we herein propose that the S-group represents a new species, which we named *M. haiboensis* sp. nov. Our findings provide a scientific basis for the targeted conservation and further research of both *M. antiquata* and *M. haiboensis* sp. nov.

## Simple Summary

This study aimed to clarify the taxonomic status of *Mactra antiquata* sensu lato, which has long been controversial due to morphological and molecular variations. Samples newly collected from four Chinese coastal provinces were analyzed using integrated morphological and molecular methods. Results showed two distinct groups (N-group and S-group) with significant differences in shell ratios, pallial sinus orientation, and escutcheon shape. Molecular analyses (phylogeny, haplotype networks, genetic distances, ASAP, and ABGD) confirmed obvious genetic differentiation exceeding intraspecific thresholds. The S-group was identified as a new species, *Mactra haiboensis* sp. nov., distributed in the East and South China Seas. This study provides diagnostic criteria for the new species and lays a foundation for its targeted conservation and further research.

## 1. Introduction

*Mactra antiquata* Spengler, 1802, commonly referred to as the short-necked clam, Xishi tongue, or surf clam, is a commercially important bivalve species in East Asia, particularly in China [1]. Historically, the supply of *Mactra antiquata* has been heavily dependent on wild capture, leading to a sharp decline in its natural population. Consequently, this species has been included in the List of Key Protected Economic Aquatic Animal and Plant Resources of China [2], with germplasm resource protection areas established in Shandong and Fujian provinces. Simultaneously, advances in artificial hatchery and breeding technologies for *Mactra antiquata* have facilitated the recovery of its resources [3]. Currently, research on *Mactra antiquata* in China is extensive, encompassing reproductive biology, aquaculture technology, and molecular biology [4,5,6]. Nevertheless, *Mactra antiquata* has a broad geographic distribution, spanning from Liaoning province in northern China to Hainan province in the south [1]. Previous studies have documented variations in both morphological and molecular traits among populations [7,8,9], prompting some scholars to hypothesize the existence of cryptic species within this taxon [10,11]. Therefore, a systematic analysis and revision of the taxonomic status of *Mactra antiquata* are imperative to support the targeted conservation of regional-specific germplasm resources.

Since its first description in 1802, the taxonomic classification of *Mactra antiquata* has been controversial, with this species synonymized with *Coelomactra antiquata* (Spengler, 1802), *Mactra chemnitzii* (Gray, 1837), *Mactra cornea* (Reeve, 1854), and *Mactra spectabilis* (Lischke, 1871) [12]. Minor differences in external morphology have been identified as one of the primary drivers of these taxonomic disputes. Lin observed significant morphological variations between two *Mactra antiquata* populations in China, i.e., the Fujian population and the Jiangsu population, reporting that the shell length/width/height ratio was 1:0.421:0.778 for the Fujian population and 1:0.479:0.821 for the Jiangsu population [13]. Consistently, Liu & Zhu also noted that the shells of *Mactra antiquata* in Jiangsu were wider than those in Fujian [14], and Lin further provided quantitative evidence, with the ratio of shell width to shell length being 0.5149 for the Jiangsu population and 0.4572 for the Fujian population [15]. In addition to morphological differences, molecular divergence among geographically distinct samples has also been reported. Due to the geographical barrier effect of the Yangtze River outflow [16], Kong et al. divided *Mactra antiquata* populations into the southern and northern clades, confirming genetic differentiation using polymorphic allozyme loci, AFLP markers, and mitochondrial *16S* rRNA [7,17,18]. Meng et al. also found the genetic differentiation between populations from Changle and Zhangzhou (both in Fujian province) and other groups, using multiple gene fragments, including *ITS1* [19], *COI* [20], *nad5* [21], *cytb* [22], and *ITS2* and *16S* [23]. Notably, the Beihai population from Guangxi province was genetically isolated from Fujian populations but clustered with northern populations in these studies. Meanwhile, comparative transcriptome [15,24] and comparative mitochondrial genomes [10,11,25] have also revealed genetic differentiation within *Mactra antiquata*, yet none of these studies included samples from Guangxi. This presents a challenge relating to the identification of *Mactra antiquata* from Guangxi. Consequently, despite multiple studies suggesting the presence of cryptic species within *Mactra antiquata* [8,11,18,25], the formal delineation and description of new species remains unresolved.

In this study, we collected *Mactra antiquata* samples from both southern and northern coastal regions of China and systematically analyzed their morphological and molecular traits. Our aims were (a) to identify distinct diagnostic characteristics among geographically separated groups and (b) to determine whether these groups represent distinct subspecies or new species.

## 2. Materials and Methods

### 2.1. Sampling

**Figure 1:**
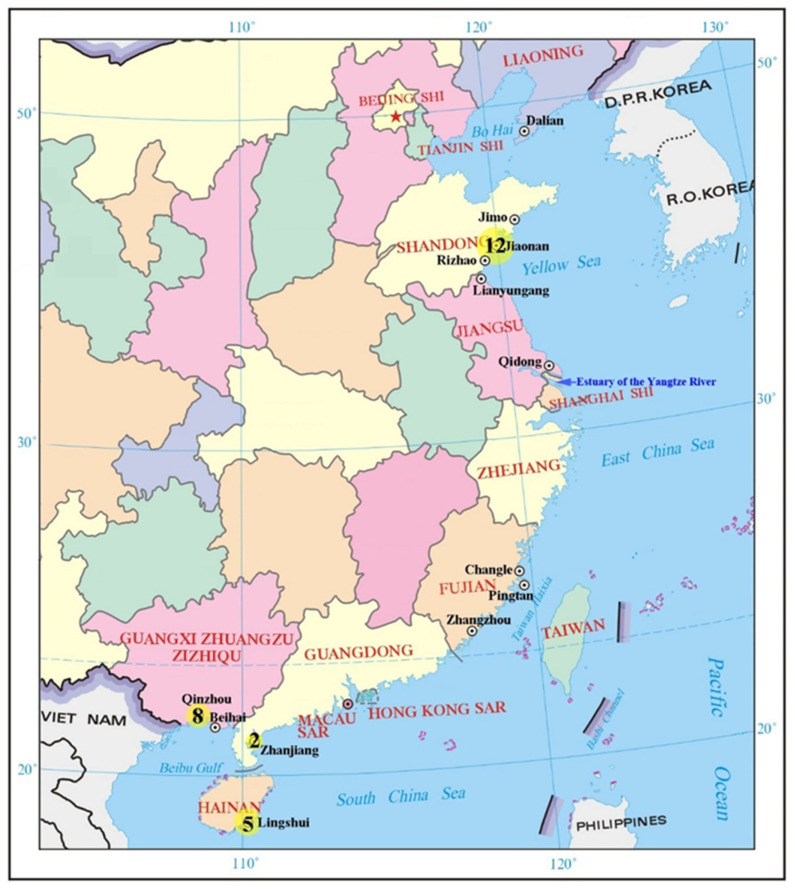
The sampling map of *Mactra antiquata* specimens in this study, with the size of the yellow circles and the figures representing the specimen quantities. The collection localities (cities in black font and provinces in red capitals) of *Mactra antiquata* sensu lato samples in China with available *COI* and *16S* rRNA sequences in GenBank are also indicated.

In this study, a total of 27 *Mactra antiquata* specimens were collected from four provinces in China, i.e., Shandong (nos. SD01-SD12), Guangxi (nos. GX01-GX08), Guangdong (nos. GD01, GD02), and Hainan (nos. HN01-HN05) (Figure 1, Table S1). All specimens were collected by hand from the intertidal zone after tidal recession and subsequently preserved in 95% ethanol immediately upon collection. Specimens collected from Shandong and Guangxi were deposited in the National Maritime Museum of China, and others were deposited in the Institute of Oceanology, Chinese Academy of Sciences. 

### 2.2. Acquisition of Morphological Characteristics

Based on the reported differences in morphological characteristics [13,14,15], the shell length, shell height, and shell width of each specimen were measured by a digital caliper (DL91150, DeLi Group Co., Ltd., Ningbo, China). In addition, other characteristics, including shell color, shell shape, umbo location, shape of adductor scar, pallial sinus, lunule, and escutcheon, etc., were described. A Canon EOS 700D camera (Canon Inc., Tokyo, Japan) was used to capture images.

### 2.3. Acquisition and Analysis of Molecular Characteristics

#### 2.3.1. DNA Extraction, PCR Amplification, and Sequencing

Total DNA of each specimen was extracted from about 30 mg of adductor muscles by the phenol–chloroform DNA isolation method [25]. Two commonly used mitochondrial genes, i.e., *COI* and *16S*, were selected for genetic identification in this study. The *COI* primer sequences were COIF-ALT: ACAAATCAYAARGAYATYGG and COIRVBSO: CCDRCNGTAAAYATRTGATG for specimens SD01 to SD12 [26] and COIF-VSA: ACCAATCATAAAGATATTGG and COIRVBSI: CCNAYHGTAAAYATATGRTG for other specimens [27]. The primers for *16S* were 16S rRNAar: CGCCTGTTTATCAAAAACAT and 16S rRNAbr: CCGGTCTGAACTCAGATCACGT [28]. All the primers were synthesized by Sangon Biotech (Shanghai, China) Co., Ltd. The PCR amplification reactions were performed using Eppendorf Mastercycler Gradient AG 22331 (Eppendorf AG, Hamburg, Germany). The amplifications for *16S* were conducted in a 25 μL system, containing 12.5 μL Taq enzyme premix, 1 μL forward primer, 1 μL reverse primer, 0.5 μL DNA template, and 10 μL ddH_2_O, while those for *COI* were in a 10 μL system, containing 5.0 μL 2 × Hieff Canace^®^PCR Master Mix (Yeasen Biotechnology (Shanghai) Co., Ltd., Shanghai, China), 0.5 μL forward primer, 0.5 μL reverse primer, 1.0 μL DNA template, and 3.0 μL ddH_2_O. The amplification protocol for *COI* sequences of specimens SD01 to SD12 followed Chen et al. [27], while the annealing temperature for *COI* of other specimens was adjusted to 43 °C. The PCR amplification program for *16S* sequences followed Yan et al. [29]. The PCR products were detected by 1.5% agarose gel electrophoresis and sequenced on a 3730XL DNA analyzer in Sangon Biotech (Shanghai, China) Co., Ltd.

#### 2.3.2. Sequence Alignment and Analysis

All new sequences were compared with sequences in GenBank using BLASTN 2.17.0+ [30,31]. Existing *COI* and *16S* sequences from GenBank (Table S2) were downloaded and aligned with sequences obtained in this study using Mega 7.0.26, and adapter sequences at both ends were deleted manually [32]. The mitochondrial genomes of *Mactra antiquata* (GenBank accession nos. KC503288, KC503290) were used to identify the location of the sequences and translated protein sequences obtained in this study by Mega 7.0.26. The *COI* and *16S* sequences of four congeneric species (*Mactra cumingii*, *Mactra chinensis*, *Mactra quadrangularis*, and *Mactra cygnus*) were extracted from their complete mitochondrial genomes (GenBank accession nos. OQ197857, OQ197855, OQ197854, and OQ197856) and used as the outgroups for phylogenetic analysis. The *COI* and *16S* sequences were aligned with MAFFT v7.505 [33] using the “--auto” strategy and normal alignment mode. Ambiguously aligned fragments were removed using Gblocks 0.91b [34]. The parameter settings for *COI* were minimum number of sequences for a conserved/flank position (28/28), maximum number of contiguous non-conserved positions (8), minimum length of a block (10), and allowed gap positions (with half), while the parameter settings for *16S* were minimum number of sequences for a conserved/flank position (30/30), maximum number of contiguous non-conserved positions (8), minimum length of a block (10), and allowed gap positions (with half). ModelFinder v2.2.0 [35] was used to select the best-fit model using the BIC criterion. The phylogenetic trees were conducted using maximum likelihood (ML) and Bayesian inference (BI) methods using PhyloSuite v1.2.3 [36,37]. Maximum likelihood phylogenies were inferred using IQ-TREE [38] under the HKY + G4 + F model for 1000 ultrafast [39] bootstraps. Bayesian inference phylogenies were inferred using MrBayes v3.2.7a [40] under the HKY + G + F model (4 parallel runs, 2.5 million generations), in which the initial 25% of sampled data were discarded as burn-in. The phylogenetic trees were visualized by iTOL v7 [41].

The genetic distances within and between the two groups based on either *COI* or *16S* sequences were estimated using the Kimura 2-parameter (K2P) model integrated into Mega 7.0.26 [32,42]. Arlequin 3.5.2.2 [43] and PopART 1.7 [44] were employed to construct the haplotype TCS networks [45]. The Assemble Species by Automatic Partitioning (ASAP) [46] and Automatic Barcode Gap Discovery (ABGD) [47] were used in this study for species delimitation in phylogenetic trees based on partial *COI* and *16S* gene sequences. The delineation results were marked in the ML trees.

Estimation of divergence times within Mactridae was conducted with a correlated rates clock in MCMCtree v4.10.9 [48]. All sequences used are listed in Table S3. The substitution models were estimated to be GTR + F + G4 by ModelFinder v2.2.0 [35]. Three fossil calibration points were selected for the divergence time estimation. The divergence time of Veneroida (416–359.2 Ma) was used as the calibration time for the root node [49]. The fossil times of *Mactra cygnus* (*Mactra alta*) (72.2~83.6 Mya) [50] and *Mulinia edulis* (0.774~1.8 Mya) [51], which are all recorded in the Paleobiology Database (https://paleobiodb.org/#/, accessed on 12 January 2026), were used for estimating divergence time calibration. A Markov chain was run for 800k generations twice and sampled every 10 generations, with the first 40% discarded as burn-in. The effective sample size (ESS) of the majority of the parameters was confirmed by Tracer v1.6 [52], which was all above 200. Figtree v1.4.3 was used to generate and visualize the time tree.

## 3. Results

### 3.1. Morphological Results

**Figure 2:**
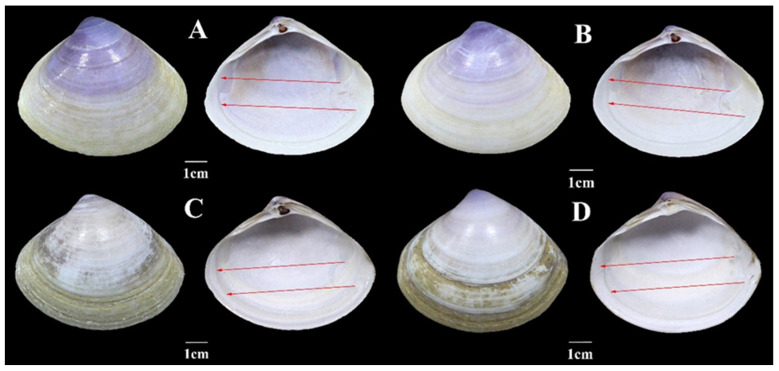
Shell morphology of *Mactra antiquata* sensu lato. (**A**) (no. GX01) and (**B**) (no. GX02) were from the S-group, while (**C**) (no. SD10) and (**D**) (no. SD11) were from the N-group. The orientation angles of the pallial sinus are marked with red arrows.

All samples in this study exhibited the following morphological characteristics (Figure 2): shell large-sized, relatively thin-walled, slightly triangular in outline; umbo prominent, inclined anteriorly, positioned at the mid-dorsal region with a slight anterior offset; lunule elongate and cordate, with indistinct margins; escutcheon lanceolate, extending the entire length of the posterior dorsal margin, surrounded by a slightly raised peripheral ridge; shell surface yellowish-white to yellowish-brown, with a purple tinge at the umbo; growth lines fine and dense, and radial ribs absent; inner surface of the shell pale purple, darkening toward the umbo; pallial sinus shallow, with a rounded apex; adductor muscle scars small; ligament groove at the hinge relatively large; cardinal teeth of the left valve zigzag-shaped, one lamellar nymph on each side; and cardinal teeth of the right valve splayed, with bilamellar nymphs on both sides.

Nevertheless, distinct morphological differences were observed between the two *Mactra antiquata* groups identified in this study. Three quantifiable traits showing significant intergroup divergence are summarized as follows. First, individuals of the S-group (collected from Guangxi, Guangdong, and Hainan provinces) had relatively narrower and shorter shells compared to those of the N-group (collected from Shandong province). Specifically, the shell width-to-length ratio of the S-group ranged from 0.456 to 0.485 (mean of 0.465), while its shell height-to-length ratio ranged from 0.750 to 0.805 (mean of 0.781). In contrast, the N-group exhibited a shell width-to-length ratio of 0.501–0.582 (mean of 0.533) and a shell height-to-length ratio of 0.795–0.852 (mean of 0.822). Secondly, the orientation angle of the pallial sinus differed between the two groups. The orientation angle was defined as the angle formed by the anterior extension along the inferior margin of the pallial sinus, with the extension width measured as the distance from the inferior margin of the intersection between the posterior adductor muscle scar and the pallial sinus to the inferior margin of the pallial sinus. For N-group individuals, the pallial sinus extended toward the region inferior to the anterior adductor muscle scar, particularly aligned with the extension line of the upper edge of the pallial sinus. In contrast, the pallial sinus of S-group individuals pointed directly toward the anterior adductor muscle scar. Third, the escutcheon of N-group individuals was significantly more slender than that of the S-group. The escutcheon length-to-width ratio (calculated as the linear distance between the two endpoints of the escutcheon relative to its width in a single valve) of the N-group ranged from 7.227 to 10.481, with a mean of 8.237, whereas that of the S-group ranged from 5.422 to 6.387, with a mean value of 5.779. Additionally, the purple pigmentation on the shells of S-group individuals was more intense, which was distinguishable even at the adductor muscle scars; however, this pigmentation trait showed high individual variability and thus was not considered a reliable diagnostic character.

### 3.2. Molecular Results

**Figure 3:**
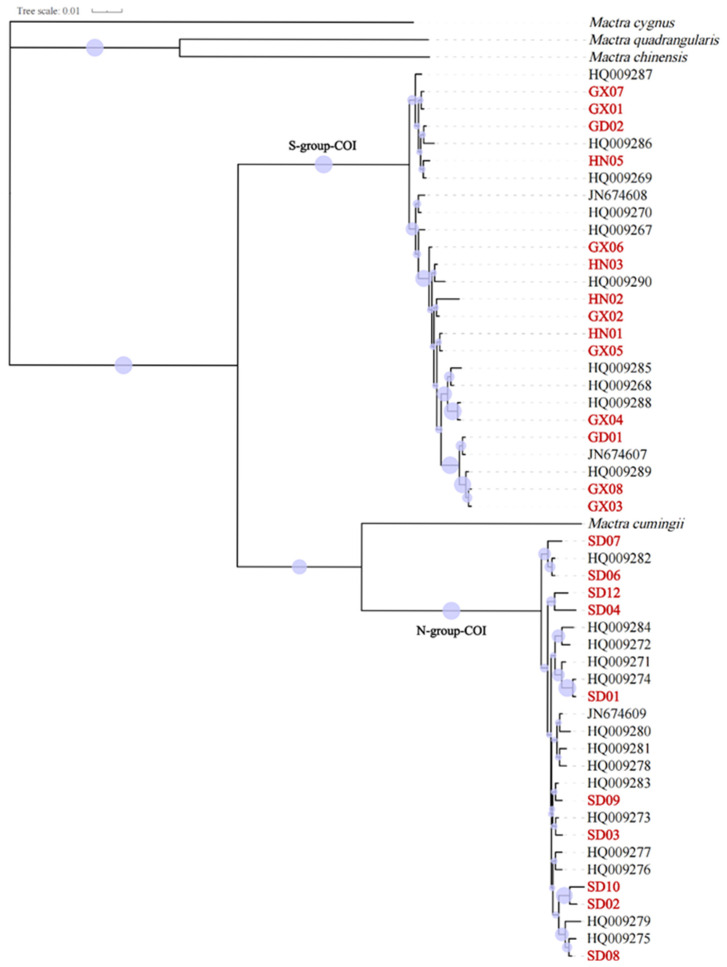
Phylogenetic tree using Bayesian inference based on partial *COI* sequences of *Mactra antiquata* sensu lato with *Mactra cumingii*, *Mactra chinensis*, *Mactra quadrangularis*, and *Mactra cygnus* being the outgroups. Newly sequenced individuals are marked in red. Purple circles display bootstraps ranging from 0 to 1.

**Figure 4:**
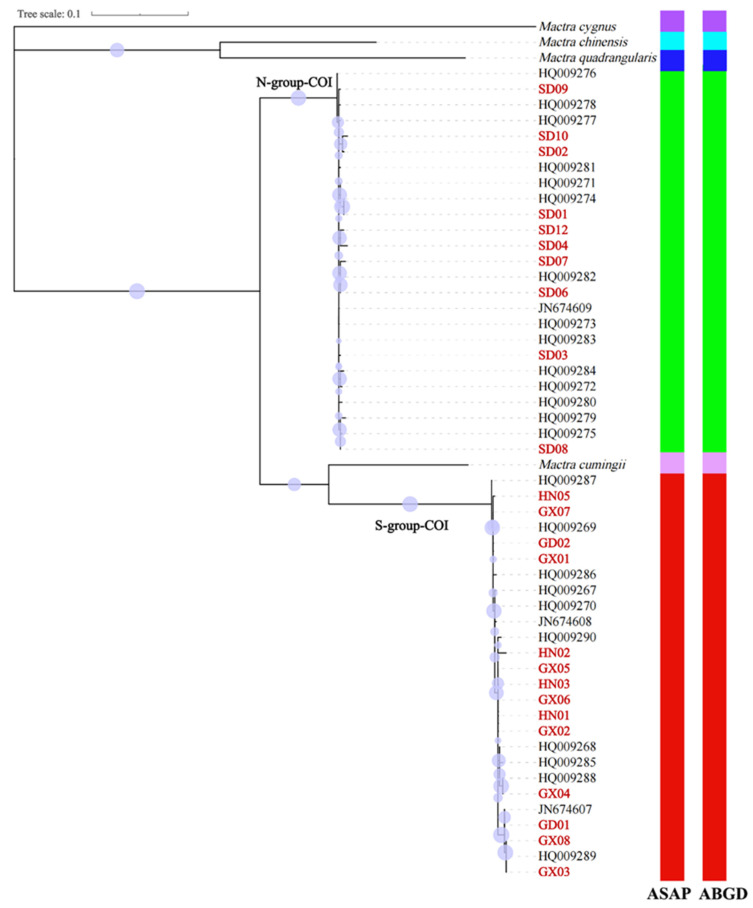
Phylogenetic tree using maximum likelihood inference based on partial *COI* sequences of *Mactra antiquata* sensu lato with *Mactra cumingii*, *Mactra chinensis*, *Mactra quadrangularis*, and *Mactra cygnus* being the outgroups. Newly sequenced individuals are marked in red. Purple circles display bootstraps ranging from 6 to 99. Results of species delimitation using ASAP and ABGD are shown, with different colors representing different species.

A total of 24 partial *COI* sequences and 27 *16S* sequences were obtained in this study. They had significant alignments with existing *Mactra antiquata* sequences (e-value 0.0 and percentage identity over 99%). After alignment, the *COI* dataset included 51 sequences of 606 bp in length, from the 115th base to the 710th base of the coding sequence of the *Mactra antiquata COI* gene. They were obviously separated into two groups, i.e., S-group-COI and N-group-COI, as shown in Figure 3 and Figure 4. The S-group-COI and N-group-COI had 26 and 25 sequences. All the sequences of S-group-COI were from individuals collected in the South China Sea (south coast of the Yangtze River) and Vietnam, including the 14 specimens in this study and other individuals previously reported. Except for four sequences from individuals collected in Beihai (HQ009274-HQ009277), other individuals whose sequences belonged to the N-group-COI were all collected from northern coastal China (the north coast of the Yangtze River). However, the ML tree showed a sister relationship of *Mactra cumingii* and S-group-COI, while the BI tree showed a sister relationship of *Mactra cumingii* with the N-group-COI. As they were not clustered directly, the genetic relationship between them was not close enough to be considered as belonging to the same species. The genetic distance between the two groups was 0.158, and within the groups, it was 0.008 for the S-group-COI and 0.006 for the N-group-COI. Almost all S-group-COI sequences had three more tryptophans encoded, located in the 86th, 108th, and 131st amino acids in the *COI* protein, than N-group-COI sequences.

**Figure 5:**
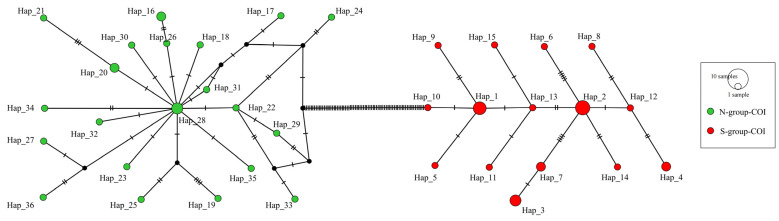
A minimum spanning tree showing the genetic relationship among 36 haplotypes of partial *COI* sequences in *Mactra antiquata* sensu lato.

A total of 36 haplotypes (GenBank accession nos. PX644086-PX644109) were detected among the 51 partial *COI* sequences (Figure 5). Among them, there were 15 haplotypes in the S-group-COI and 21 haplotypes in the N-group-COI. Eight *COI* haplotypes were shared by two (Hap_4, Hap_7, Hap_16, and Hap_20) or more specimens (Hap_1, Hap_2, Hap_3, and Hap_28). The number of individuals with haplotype 2 was the largest, amounting to 5, followed by Hap_1 (4 individuals).

**Figure 6:**
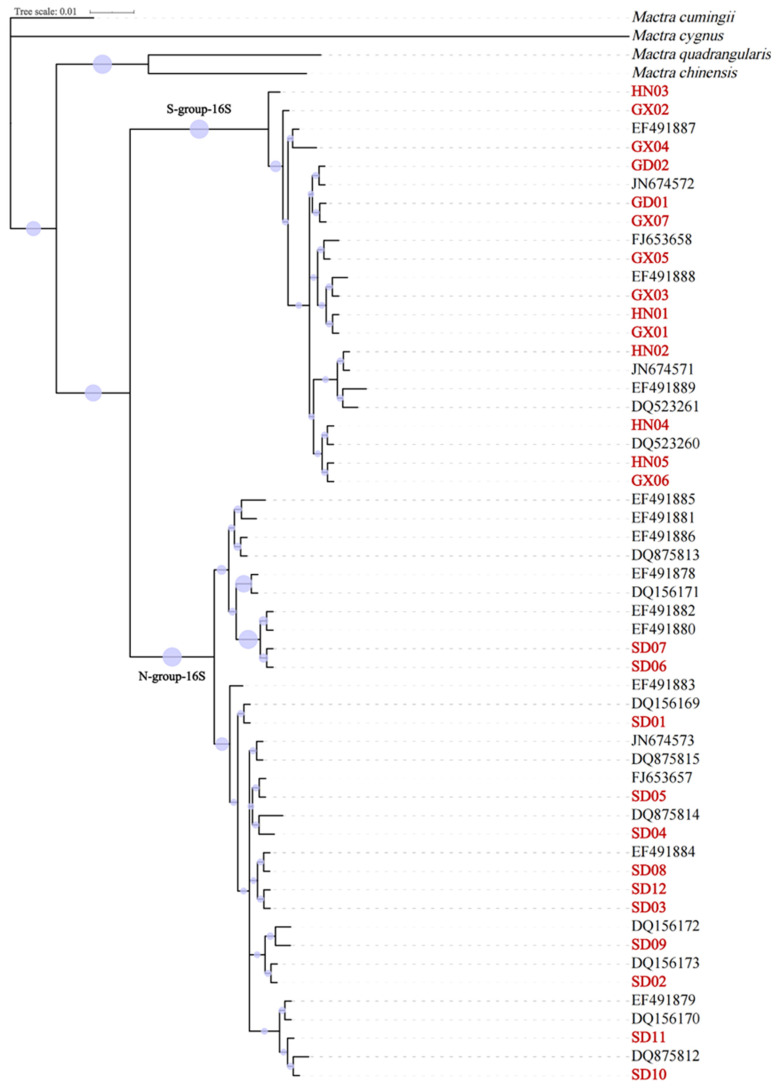
Phylogenetic tree using Bayesian inference based on partial *16S* sequences of *Mactra antiquata* sensu lato with *Mactra cumingii*, *Mactra chinensis*, *Mactra quadrangularis*, and *Mactra cygnus* being the outgroups. Newly sequenced individuals are marked in red. Purple circles display bootstraps ranging from 0 to 1.

**Figure 7:**
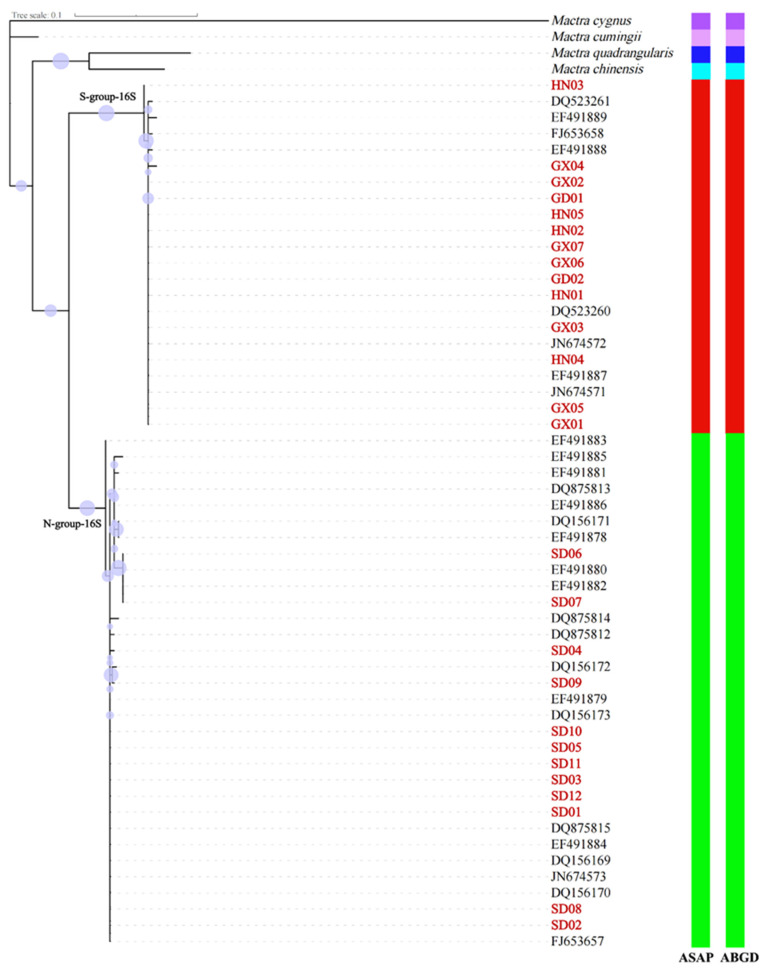
Phylogenetic tree using maximum likelihood inference based on partial *16S* sequences of *Mactra antiquata* sensu lato with *Mactra cumingii*, *Mactra chinensis*, *Mactra quadrangularis*, and *Mactra cygnus* being the outgroups. Newly sequenced individuals are marked in red. Purple circles display bootstraps ranging from 18 to 96. Results of species delimitation using ASAP and ABGD are shown, with different colors representing different species.

For *16S*, the dataset included 54 sequences of 306 bp in length, from the 686th base to the 991st base of the complete *16S* ribosomal RNA of *Mactra antiquata*. The phylogenetic trees based on partial *16S* sequences using maximum likelihood and Bayesian inference methods were not consistent with each other, nor with those based on partial *COI* sequences (Figure 6 and Figure 7). The *16S* sequences of *Mactra antiquata* were split into two branches, i.e., S-group-16S and N-group-16S. The S-group-16S and N-group-16S had 22 and 32 sequences, respectively. Similar to the results based on *COI*, all the sequences of S-group-16S were from individuals collected in the South China Sea. Except for two sequences from individuals collected in Beihai (DQ875812 and DQ875813), other individuals whose sequences belonged to the N-group-16S were all collected from northern coastal China. Both trees showed a sister relationship between the two groups. The genetic distance between the groups was 0.084, and within the groups, it was 0.002 for the S-group-16S and 0.005 for the N-group-16S. It is interesting to note that all individuals from Shandong in this study had different haplotypes.

**Figure 8:**
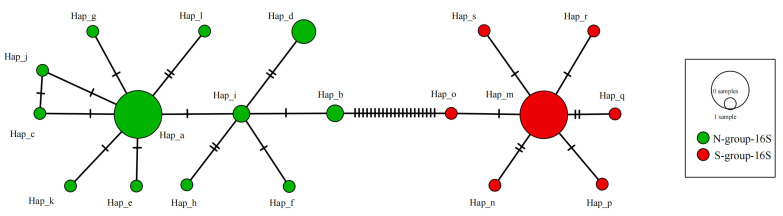
A minimum spanning tree showing the genetic relationship among 19 haplotypes of partial *16S* sequences in *Mactra antiquata* sensu lato.

A total of 19 haplotypes (GenBank accession nos. PX643058-PX643083) were detected among the 54 partial *16S* sequences (Figure 8). Among them, there were 7 haplotypes in the S-group-16S and 12 haplotypes in the N-group-16S. Only five *16S* haplotypes were shared by multi-specimens (Hap_a, Hap_b, Hap_d, Hap_i, and Hap_m). Both Hap_a and Hap_m had the largest number of individuals (16). Interestingly, 12 individuals from all 14 sequences exhibited Hap_m (Table S1). Except for Hap_m, Hap_n, and Hap_o, no other haplotypes were found among the samples in this study. Overall, no haplotypes were shared between the two groups for either *COI* or *16S*. There were 106 and 37 polymorphic loci for *COI* and *16S*, respectively. Both minimum spanning trees exhibited a large difference between individuals of the S-group and the N-group.

Based on 51 partial *COI* sequences, both the first- and second-ranked ASAP results identified six species with ASAP scores of 1.0 and 2.5, respectively. Both results identified *Mactra antiquata* as two distinct species (Figure 4). The ABGD showed the same species delimitation with prior intraspecific divergence values of 0.0046 to 0.0599. Based on 54 partial *16S* sequences, the first-ranked ASAP result identified three species with an ASAP score of 1.5 but identified *Mactra cumingii*, *Mactra chinensis*, *Mactra quadrangularis*, and *Mactra cygnus* as one species (Figure 7). All the results identified *Mactra antiquata* sequences as two or more species. Among them, the second-ranked result identified six species with an ASAP score of 2.5, with *Mactra antiquata* identified as two species. All ten results by ABGD were consistent with the second ASAP result.

**Figure 9:**
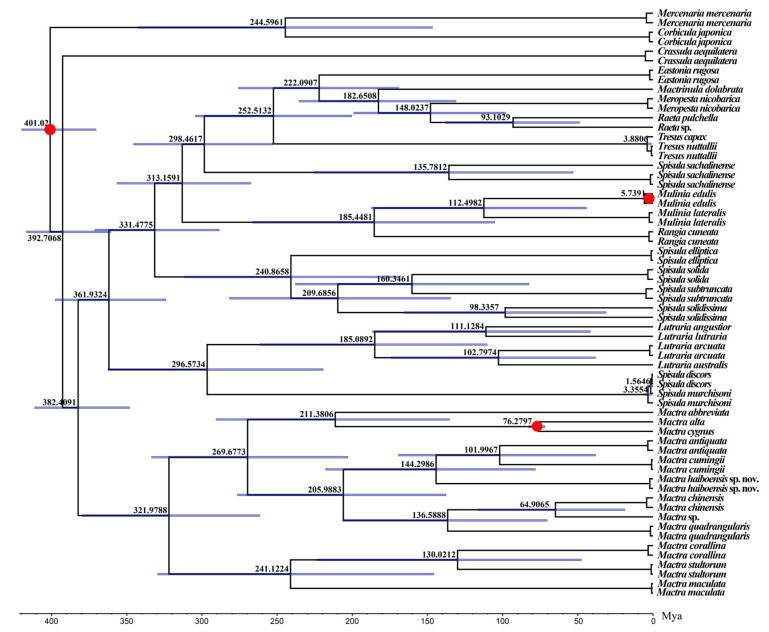
Estimates of divergence times within the family Mactridae based on combining partial *COI* and *16S* sequences. Numbers near the nodes indicate the median ages, and blue bars indicate 95% highest posterior density intervals. Calibration points are marked using red circles.

The phylogenetic tree constructed based on the combined *COI* and *16S* gene sequences showed that *Mactra antiquata* of the N-group was closely clustered with *Mactra cumingii* and then grouped into a single clade with *Mactra antiquata* of the S-group (i.e., *Mactra haiboensis* in Figure 9). The divergence time between *Mactra antiquata* of the N-group and *Mactra cumingii* was 102.00 Mya, leading to the formation of two independent species. In contrast, *Mactra antiquata* of the S-group originated earlier, with a divergence time of 144.30 Mya from the aforementioned two species.

## 4. Discussion

### 4.1. Identification of Cryptic Species in Mactra antiquata Sensu Lato

Although the morphological characteristics of *Mactra antiquata* sensu lato were generally similar, subtle differences could be identified upon careful observation. In this study, size-related traits allowed the clear delineation of samples into two groups (N-group and S-group), which was consistent with the findings of Liu & Zhu [13], Lin [14], and Qu [15]. Specifically, the N-group and S-group in this study corresponded to the Jiangsu population and Fujian population reported in previous studies, respectively. Our results provide additional morphological diagnostic criteria, among which the orientation of the pallial sinus is particularly valuable, as it enables the differentiation of the two groups without the need for quantitative measurement. As a key taxonomic trait, the shape of the pallial sinus is also widely used as an identification criterion for species of *Meretrix*, a genus with highly similar external morphology [53]. Furthermore, Liu & Zhu measured the width and length of the escutcheon [13] but did not calculate the length-to-width ratio. Our study revealed significant intergroup differences in escutcheon morphology (reflected by the length-to-width ratio), which can serve as a reliable taxonomic criterion for distinguishing the two groups.

Although the phylogenetic trees based on *COI* and *16S* exhibited slight discrepancies in the topological relationships between the two groups of *Mactra antiquta* sensu lato and *Mactra cumingii*, clear genetic differentiation between the two groups was consistently observed. This genetic relationship based on partial *COI* sequences is consistent with that reconstructed using 12 mitochondrial protein-coding genes and two mitochondrial rRNAs [11], while our result based on *16S* aligned with the Bayesian tree inferred from combined *COI* and *16S* sequences [8]. Ni defined that the range of K2P genetic distances among individuals within the same species and among species within the same genus of *Mactra* calculated based on the COI gene sequences were 0.000–0.021 (with a mean value of 0.006) and 0.085–0.284 (with a mean value of 0.209), respectively, while they were 0.000–0.009 (with a mean value of 0.002) and 0.014–0.271 (with a mean value of 0.182) based on *16S* gene sequences [54]. We found the genetic distances between the two groups of *Mactra antiquata* sensu lato were 0.158 and 0.084 based on *COI* and *16S* sequences, respectively. The results exceeded the intraspecific genetic distance while remaining within the interspecific genetic distance range proposed by Ni [54]. Collectively, these molecular data strongly support the existence of cryptic species within *Mactra antiquata* sensu lato, consistent with previous studies [8,11,20,23].

### 4.2. Identification of New Species in Mactra antiquata Sensu Lato

Since Spengler established the name *Mactra antiquata* in 1802, this taxon has accumulated several synonyms, including *Coelomactra antiquata* (Spengler, 1802), *Mactra chemnitzii* (Gray, 1837), *Mactra cornea* (Reeve, 1854), and *Mactra spectabilis* (Lischke, 1871) [12]. Beyond the genus name revision (from *Mactra* to *Coelomactra* for one synonym), both *Mactra chemnitzii* and the original *Mactra antiquata* were described based on the specimen misidentified as *Mactra violacea australis* by Chemnitz, according to their original taxonomic descriptions. Quantitative analysis of the illustrations of the type material showed that the shell height-to-length ratio of the individual depicted by Chemnitz [55] was 0.835. In addition, the specimens illustrated by Reeve in 1854 and photographed by Lischke in 1871 had ratios of shell height to shell length of 0.823 and 0.829, respectively. These values were consistent with the range of the N-group in our study (shell height-to-length ratio ranged from 0.795 to 0.852, with a mean value of 0.822), supporting the inclusion of all these type specimens (from original descriptions) within the N-group. Although none of these historical descriptions provided views of the inner valve (where key traits such as pallial sinus orientation reside), the consistent shell morphometric evidence further confirms that the S-group specimens represent a new species.

Based on the *COI* sequences, all individuals collected from the Yellow Sea and Bohai Sea were clustered within the N-group, except for four specimens previously documented as collected from Beihai, Guangxi province [20]. In contrast, all S-group specimens were collected in the East China Sea and South China Sea. Although no specimens were collected from Fujian province or Vietnam in this study, where *Mactra antiquata* samples were proposed as a new species, our results indicate that these uncollected populations are conspecific with the S-group specimens identified herein. Thus, the current known distribution range of the new species can be inferred to include Guangxi, Guangdong, Fujian, and Hainan provinces (China) and Vietnam. This species exhibited better warm-water adaptability compared with those from the N-group. In this study, we formally named this new species as *Mactra haiboensis* sp. nov.

### 4.3. Description of Mactra haiboensis sp. nov.

Taxonomy

Order—Venerida Gray, 1854

Superfamily—Mactroidea Lamarck, 1809

Family—Mactridae Lamarck, 1809

Subfamily—Mactrinae Lamarck, 1809

Genus—*Mactra* Linnaeus, 1767

Species—*Mactra haiboensis* sp. nov. Ma, Teng, Liu, & Wang

Etymology: The specific epithet honors this species as the first new taxon discovered by the National Maritime Museum of China. Its Chinese name is “海博蛤蜊”.

Holotype: GX01. The specimen was collected on 16 November 2023 and deposited in the National Maritime Museum of China.

Type locality: Sanniangwan, Qinzhou, Guangxi, China.

Paratypes: GX02–GX08. Details are shown in Table S1. Specimens were deposited in the National Maritime Museum of China.

Diagnostic features: Ratio of shell width to shell length around 0.465, ratio of shell height to shell length around 0.781, pallial sinus pointing to the anterior adductor scar, and escutcheon wider and purple area larger than *Mactra antiquata*.

Description: Shell large-sized, relatively thin-walled, slightly triangular in outline; umbo prominent, anteriorly inclined, positioned at the mid-dorsal region with a slight anterior offset; lunule elongate, cordate, with indistinct margins; escutcheon lanceolate, extending the entire length of the posterior dorsal margin, surrounded by a slightly raised peripheral ridge; shell surface yellowish-brown, with a purple tinge at the umbo; growth lines fine and dense, radial ribs absent; inner surface of the shell pale purple, darkening toward the umbo; pallial sinus shallow, with a rounded apex, directed directly toward the anterior adductor muscle scar; adductor muscle scars small; ligament groove at the hinge relative large; cardinal teeth of the left valve zigzag-shaped, with one lamellar nymph on each side; and cardinal teeth of the right valve splayed, with bilamellar nymphs on both sides.

Habitat: Sandy area of the low intertidal zones and a depth of 20 m underwater.

Sampling distribution: Fujian, Guangxi, Guangdong, Hainan, Vietnam.

## 5. Conclusions

In this study, we confirmed the existence of a new species within *Mactra antiquata* sensu lato through an integrated analysis of morphological and molecular evidence and formally named it *Mactra haiboensis* sp. nov. The currently known distribution range of the new species covers the East China Sea and South China Sea, including four provinces in China (Fujian, Guangdong, Guangxi, and Hainan) and Vietnam. We also proposed three simple and practical morphological methods for distinguishing *Mactra haiboens* sp. nov. from *Mactra antiquata* sensu stricto. Genetic differentiation between the two species was comprehensively verified by phylogenetic analysis, haplotype network construction, genetic distance calculation, and species delimitation methods (ASAP and ABGD). Additionally, previous studies on comparative gene fragments, transcriptomes, and mitochondrial genomes provide supplementary evidence that merits further verification to reinforce our conclusions.

## Data Availability

Data are contained within the article and Supplementary Materials.

## Supplementary Materials

The following supporting information can be downloaded at https://www.mdpi.com/article/10.3390/biology15020178/s1, Table S1: Collection and sequence information of newly sequenced *Mactra antiquata* sensu lato; Table S2: Existing *COI* and *16S* sequences of *Mactra antiquata* sensu lato in GenBank; Table S3: *COI* and *16S* genes downloaded from GenBank for phylogenetic analyses.
