# Supplementary material for: Morphological and Molecular Evidence Revealed New Species in Mactra antiquata Sensu Lato
Source: Biology (Basel). 2026 Jan 18;15(2):178. doi: 10.3390/biology15020178 (PMC12837493; doi:10.3390/biology15020178)
Supplement: Supplementary file 1 [file biology-15-00178-s001.zip › biology-4054308-supplementary.pdf]

Table S1. Collection and sequence information of newly sequenced *Mactra antiquata sensu lato*

Number	Locality	Voucher No.	Colletion date	Sequence obtained	
				COI	*16S*
GX01	Sanniangwan, Qinzhou, Guangxi	GHB0015201	16 Nov. 2023	Hap_1	Hap_m
GX02	Sanniangwan, Qinzhou, Guangxi	GHB0015202	16 Nov. 2023	Hap_2	Hap_m
GX03	Sanniangwan, Qinzhou, Guangxi	GHB0015203	16 Nov. 2023	Hap_3	Hap_m
GX04	Sanniangwan, Qinzhou, Guangxi	GHB0015204	16 Nov. 2023	Hap_4	Hap_n
GX05	Sanniangwan, Qinzhou, Guangxi	GHB0015205	16 Nov. 2023	Hap_2	Hap_m
GX06	Sanniangwan, Qinzhou, Guangxi	GHB0015206	16 Nov. 2023	Hap_2	Hap_m
GX07	Sanniangwan, Qinzhou, Guangxi	GHB0015207	16 Nov. 2023	Hap_1	Hap_m
GX08	Sanniangwan, Qinzhou, Guangxi	GHB0015208	16 Nov. 2023	Hap_3	/
HN01	Lianwan, Lingshui, Hainan	MA147	11 Dec. 2020	Hap_2	Hap_m
HN02	Lianwan, Lingshui, Hainan	MA148	11 Dec. 2020	Hap_6	Hap_m
HN03	Lianwan, Lingshui, Hainan	MA149	11 Dec. 2020	Hap_2	Hap_o
HN04	Lianwan, Lingshui, Hainan	MA150	11 Dec. 2020	/	Hap_m
HN05	Lianwan, Lingshui, Hainan	MA151	11 Dec. 2020	Hap_5	Hap_m
GD01	Donghai island, Zhanjiang, Guangdong	MA131	27 Nov. 2020	Hap_7	Hap_m
GD02	Donghai island, Zhanjiang, Guangdong	MA132	27 Nov. 2020	Hap_1	Hap_m
SD01	Shandongtou, Qingdao, Shandong	GHB0015209	10 Mar. 2025	Hap_16	Hap_a
SD02	Shandongtou, Qingdao, Shandong	GHB0015210	10 Mar. 2025	Hap_17	Hap_a
SD03	Shandongtou, Qingdao, Shandong	GHB0015211	10 Mar. 2025	Hap_18	Hap_a
SD04	Shandongtou, Qingdao, Shandong	GHB0015212	10 Mar. 2025	Hap_19	Hap_e
SD05	Shandongtou, Qingdao, Shandong	GHB0015213	10 Mar. 2025	/	Hap_a
SD06	Shandongtou, Qingdao, Shandong	GHB0015214	10 Mar. 2025	Hap_20	Hap_d
SD07	Shandongtou, Qingdao, Shandong	GHB0015215	10 Mar. 2025	Hap_21	Hap_d
SD08	Shandongtou, Qingdao, Shandong	GHB0015216	10 Mar. 2025	Hap_22	Hap_a

SD09	Shandongtou, Qingdao, Shandong	GHB0015217	10 Mar. 2025	Hap_23	Hap_c
SD10	Shandongtou, Qingdao, Shandong	GHB0015218	10 Mar. 2025	Hap_24	Hap_a
SD11	Shandongtou, Qingdao, Shandong	GHB0015219	10 Mar. 2025	/	Hap_a
SD12	Shandongtou, Qingdao, Shandong	GHB0015220	10 Mar. 2025	Hap_25	Hap_a

Table S2. Existing *COI* and *16S* sequences of *Mactra antiquata sensu lato* in GenBank

Locality	*COI*			*16S*			References
	Voucher No.	Accession No.	Haplotype no. in this study	Voucher No.	Accession No.	Haplotype no. in this study	
Changle, Fujian	X1	JN674607	Hap_7	X1	JN674571	Hap_m	Ni et al. 2012
Pingtan, Fujian	X2	JN674608	Hap_15	X2	JN674572	Hap_m	
Lianyungang, Jiangsu	X3	JN674609	Hap_29	X3	JN674573	Hap_a	
Jimo, Shandong				JM27	EF491878	Hap_b	Kong et al. 2009
Jimo, Jiaonan, and Rizhao, Shandong; Qidong, Jiangsu				JM28	EF491879	Hap_a	
Jimo, Shandong				JM33	EF491880	Hap_d	
Jiaonan, Shandong				JN11	EF491881	Hap_f	
Jiaonan, Shandong				JN14	EF491882	Hap_d	
Jiaonan, Shandong				JN15	EF491883	Hap_g	
Rizhao, Shandong; Qidong, Jiangsu				QD2	EF491884	Hap_a	
Qidong, Jiangsu				QD7	EF491885	Hap_h	
Qidong, Jiangsu				RZ5	EF491886	Hap_i	

Changle and Zhangzhou, Fujian				CL5	EF491887	Hap_m	
Changle, Fujian				CL10	EF491888	Hap_q	
Changle, Fujian				CCL11	EF491889	Hap_r	
Changle, Fujian				CL9	DQ523261	Hap_s	Meng et al. 2013
Beihai, Guangxi				GX7	DQ875812	Hap_k	
Lianyungang, Jiangsu				LYG4	DQ875814	Hap_l	
Qidong, Jiangsu				QD	DQ156171	Hap_b	
Qidong, Jiangsu				QD1	DQ156172	Hap_j	
Beihai, Guangxi				GX2	DQ875813	Hap_i	
Changle, Fujian				CL2	DQ523260	Hap_m	
Lianyungang, Jiangsu				LYG	DQ156169	Hap_a	
Lianyungang, Jiangsu				LYG1	DQ875815	Hap_a	
Jiaonan, Shandong				JD	DQ156170	Hap_a	
Qidong, Jiangsu				XM	DQ156173	Hap_a	
				QD12	FJ653657	Hap_a	Chen et al. (Unpublished)
				CL72	FJ653658	Hap_p	Chen et al. (Unpublished)
Changle, Fujian	CL1	HQ009267	Hap_11				Meng et al. 2011
Changle, Fujian	CL4	HQ009268	Hap_12				
Changle, Fujian	CL8	HQ009269	Hap_1				

Changle, Fujian	CL10	HQ009270	Hap_13
Dalian, Liaoning	DL2	HQ009271	Hap_26
Dalian, Liaoning	DL4	HQ009272	Hap_27
Dalian, Liaoning	DL12	HQ009273	Hap_28
Beihai, Guangxi	GX1	HQ009274	Hap_16
Beihai, Guangxi	GX8	HQ009275	Hap_29
Beihai, Guangxi	GX23	HQ009276	Hap_30
Beihai, Guangxi	GX24	HQ009277	Hap_31
Qidong, Jiangsu	QD1	HQ009278	Hap_32
Qidong, Jiangsu	QD4	HQ009279	Hap_33
Qidong, Jiangsu	QD7	HQ009280	Hap_34
Qidong, Jiangsu	QD8	HQ009281	Hap_35
Rizhao, Shandong	RZ1	HQ009282	Hap_20
Rizhao, Shandong	RZ5	HQ009283	Hap_29
Shandong	SD2	HQ009284	Hap_36
Vietnam	YN1	HQ009285	Hap_8
Vietnam	YN3	HQ009286	Hap_9
Vietnam	YN5	HQ009287	Hap_10
Zhangzhou, Fujian	ZZ1	HQ009288	Hap_4
Zhangzhou, Fujian	ZZ3	HQ009289	Hap_3

Zhangzhou, Fujian	ZZ5	HQ009290	Hap_14

Table S3. *Cox1* and *16S* genes downloaded from GenBank for phylogenetic analyses.

Species	*cox1* Accession	*16S* Accession
Mactra antiquata	SD01#	SD01#
Mactra antiquata	SD02#	SD02#
Mactra haiboensis sp. nov.	GX01#	GX01#
Mactra haiboensis sp. nov.	GX02#	GX02#
Mactra cumingii	JN674610	JN674574
Mactra cumingii	(OQ197857)	(OQ197857)
Mactra chinensis	KJ754823	JN674595
Mactra chinensis	(OQ197855)	(OQ197855)
Mactra quadrangularis	(OQ197854)	(OQ197854)
Mactra quadrangularis	KC205732	JN674585
Mactra cygnus	(OQ197856)	(OQ197856)
Mactra alta	JN674617	JN674580
Mactra abbreviata	MN696998	
Mactra corallina	KM673272	FJ830416
Mactra corallina	KM673273	FJ830417
Mactra stultorum	KR084430	
Mactra stultorum	KR084822	
Mactra maculata	JN674613	JN674577
Mactra maculata	JN674614	JN674578
Mactra sp.	(MT780813)	(MT780813)
Spisula murchisoni	HQ575230	

Spisula murchisoni	HQ575229C	
Spisula discors	HQ575231	
Spisula discors	HQ575233	
Spisula sachalinense	MN119643	KP090053
Spisula sachalinense	MN119626	KP090054
Spisula sachalinense	(MG432821)	(MG432821)
Spisula elliptica	KR084658	
Spisula elliptica	KR084643	
Spisula solidissima	AY707805	
Spisula solidissima	AY707809	
Spisula subtruncata	MG935351	AJ548774
Spisula subtruncata	KR084884	
Spisula solida	KR084715	KX713257
Spisula solida	KR084630	JF808191
Crassula aequilatera	HM888029	
Crassula aequilatera	HM888026	
Rangia cuneata	MK878617	KT959495
Rangia cuneata	KT959383	KC429310
Mulinia edulis	JF301800	
Mulinia edulis	JF301799	
Mulinia lateralis	MN326303	MN176699
Mulinia lateralis	KT959381	MK026967
Lutraria angustior	MN064618	
Lutraria lutraria	KR084641	
Lutraria australis	JN674600	JN674564

Lutraria arcuata	JN674603	JN674565
Lutraria arcuata	JN674601	JN674566
Tresus capax	KF643926	KC429311
Tresus nuttallii	JN133754	JN133687
Tresus nuttallii	JN133753	JN133688
Eastonia rugosa	KM673287	
Eastonia rugosa	KM673286	
Meropesta nicobarica	KX713478	KX713234
Meropesta nicobarica	JN674606	JN674570
Mactrinula dolabrata	(OQ197858)	(OQ197858)
Raeta sp.	(OQ197859)	(OQ197859)
Raeta pulchella	(OQ197853)	(OQ197853)
Corbicula japonica	AB845593*	AB522669*
Corbicula japonica	MN119644*	AB522666*
Mercenaria mercenaria	HM124618*	AB236177*
Mercenaria mercenaria	HM124619*	AB236178*

Accession numbers marked with # were sequenced in this study, with * were used as outgroups, with () were extracted from mitochondrial genomes from GenBank.
